# Improving the management of imported schistosomiasis haematobia in China: lessons from a case with multiple misdiagnoses

**DOI:** 10.1186/1756-3305-6-260

**Published:** 2013-09-11

**Authors:** Hai-Yong Hua, Wei Wang, Guo-Qun Cao, Feng Tang, You-Sheng Liang

**Affiliations:** 1 Jiangsu Institute of Parasitic Diseases, 117 Yangxiang, Meiyuan, Wuxi City, Jiangsu Province 214064, People’s Republic of China; 2 Key Laboratory on Technology for Parasitic Disease Prevention and Control, Ministry of Health, 117 Yangxiang, Meiyuan, Wuxi City, Jiangsu Province 214064, People’s Republic of China; 3 Jiangsu Provincial Key Laboratory of Molecular Biology of Parasites, 117 Yangxiang, Meiyuan, Wuxi City, Jiangsu Province 214064, People’s Republic of China

**Keywords:** *Schistosoma haematobium*, Imported case, Case report, China

## Abstract

**Background:**

Human *Schistosoma haematobium* infection that causes urinary schistosomiasis occurs in Africa and the eastern Mediterranean, and China is only endemic for *S. japonicum.* In this report, we reported an imported case with *S. haematobium* infection returning from Angola to Shaanxi Province, northwestern China, where *S. japonicum* is not endemic.

**Findings:**

The case was misdiagnosed as ureteral calculus, invasive urothelial carcinoma and eosinophilic cystitis in several hospitals, and was finally diagnosed by means of serological assay followed by microscopic examination of the urine sediment. The patient was then treated with praziquantel, and a satisfactory outcome was obtained.

**Conclusions:**

As *S. haematobium* is not indigenous to China, most Chinese doctors and medical technicians are unfamiliar with this introduced parasitic disease, therefore, they need to increase the awareness of its existence when they encounter persons who have visited or resided in endemic areas, and the techniques for detection of the parasite, so as to reduce the misdiagnosis. In addition, health education should be given to those who will go to the endemic areas to improve their knowledge and awareness on prevention and control of schistosomiasis haematobia, thereby reducing the risk of exposure to the infested freshwater.

## Findings

Among the global parasitic diseases, schistosomiasis ranks second behind malaria in terms of socio-economic and public health importance in tropical and subtropical areas of the world [1]. This neglected tropical disease is estimated to be endemic in 76 countries of the developing world, which affects 300 million people, with a further 779 million at risk of infection [2]. Three major schistosome species are known to infect humans, including *Schistosoma haematobium* that causes urinary schistosomiasis which occurs in Africa and the eastern Mediterranean, *S. mansoni* that is endemic in Africa, the eastern Mediterranean, the Caribbean, and South America, and *S. japonicum* that is endemic mainly in China and the Philippines [3,4]. China is only endemic for *S. japonicum*. However, the increasing globalization of the world economy and population migration makes the introduction of *S. haematobium* and *S. mansoni* infection possible into the non-endemic areas [5]. Currently, an estimated 10 million Chinese people are working in African countries each year, and they usually stay there for several years [6]. These workers usually lack knowledge and awareness on prevention and control of schistosomiasis haematobia, resulting in the possibility of exposure to *S. haematobium*-infested freshwater in endemic regions. In addition, these migrant workers are often misdiagnosed if they are infected in Africa and have clinical symptoms after returning to their original residence in China, due to scarce knowledge and awareness of schistosomiasis haematobia prevention and control [7]. In this study, we reported a case with *S. haematobium* infection that was misdiagnosed as ureteral calculus, invasive urothelial carcinoma and eosinophilic cystitis in several hospitals. This study was approved by Jiangsu Institute of Parasitic Diseases and Key Laboratory on Technology for Parasitic Disease Prevention and Control, Ministry of Health. An informed consent was obtained from the patient, following a detailed description of the potential benefits of the study.

A 36-year-old man from Qianxian County of Shaanxi Province, China worked in Luanda, Angola during the period from July 2007 to March 2011, due to export of labor services. Before moving to Angola, his living and working areas were limited in Shaanxi Province where schistosomiasis japonica is not endemic. The man returned to Shaanxi Province between July and September, 2009. During the stay in Africa, he was frequently in contact with local rivers due to the need of living and production.

**Figure 1:**
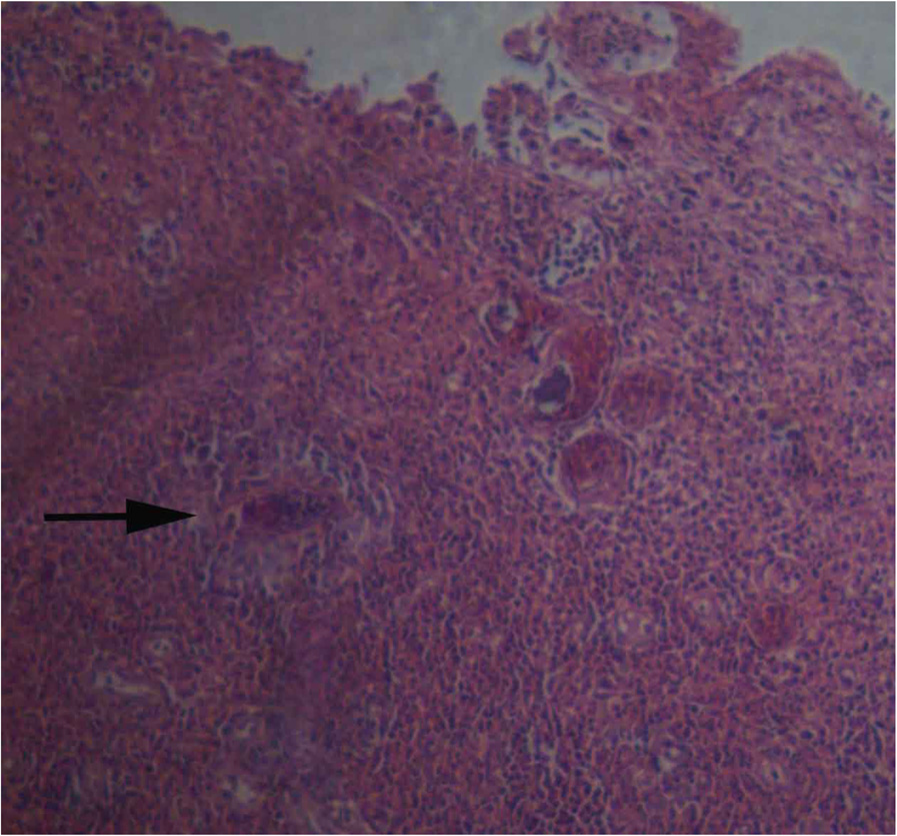
***S. haematobium *egg seen in the bladder specimen.**

In early 2010, the man accidentally found a white membrane-like substance in the urine, with a size of about 5 mm, and he did not see a doctor due to absence of subjective symptoms. On July 2010, the case was presented to a local hospital aided by China with a complaint of few streaks of blood in the urine, and he was diagnosed as ureteral calculus. However, hematuria still occurred occasionally following treatment. After returning from Angola to China on March 2011, he was admitted to Xijing Hospital Affiliated to the Fourth Military Medical University (Xi’an, China), and B ultrasonography revealed no obvious abnormality. The case received medical care in the Central Hospital of China Railway 20 Bureau Group Corporation (Xianyang, China) due to persistent presence of gross hematuria on November 16, 2011, and cystoscopy showed foreign bodies in the right posterior wall of the bladder, and invasive urothelial carcinoma with local granulomatous reactions was diagnosed by the pathology. He underwent transurethral bladder tumor resection in Xijing Hospital on November 22, 2011, and the biopsy pathology revealed eosinophilic cystitis with local urothelial hyperplasia. A routine blood test on November 25, 2011 showed a white blood cell (WBC) count of 6.75 × 10^9^/L, and 12% eosinophil granulocytes. After discharge from the hospital, the case was treated by perfusion with pirarubicin for 8 times. The cystoscopic re-examination on February 27, 2012 in Xijing Hospital showed new foreign bodies in the right posterior wall of the bladder, and the pathological examination revealed chronic bladder inflammation complicated by eosinophilic abscess and granuloma, with schistosome eggs seen (Figure 1). Serological examination was suggested for the detection of schistosome infection, and the case was treated with praziquantel with a total dose of 600 mg/kg divided TID for 3 successive days since March 3, 2012. A routine blood test on March 8, 2012 in Xijing Hospital showed a WBC count of 6.52 × 10^9^/L, and 11.2% eosinophil granulocytes. On March 13, 2012, the follow-up showed that the patient still had painless hematuria and white membrane-like substances in the urine following praziquantel treatment, and three serological tests for the detection of antibodies against *S. japonicum*, including dipstick dye immunoassay (DDIA) [8], indirect haemagglutination assay (IHA; titer, 1:40) [9] and circumoral precipitin test (COPT) [10], were all positive for schistosomes, conducted in Jiangsu Institute of Parasitic Diseases (Wuxi, China). The case was therefore diagnosed as schistosomiasis haematobia, and was then given praziquantel treatment at a total dose of 900 mg/kg divided BID for 2 successive days. During this period, the patient had no other symptoms except intermittent painless hematuria, and he had a stable body weight.

**Figure 2:**
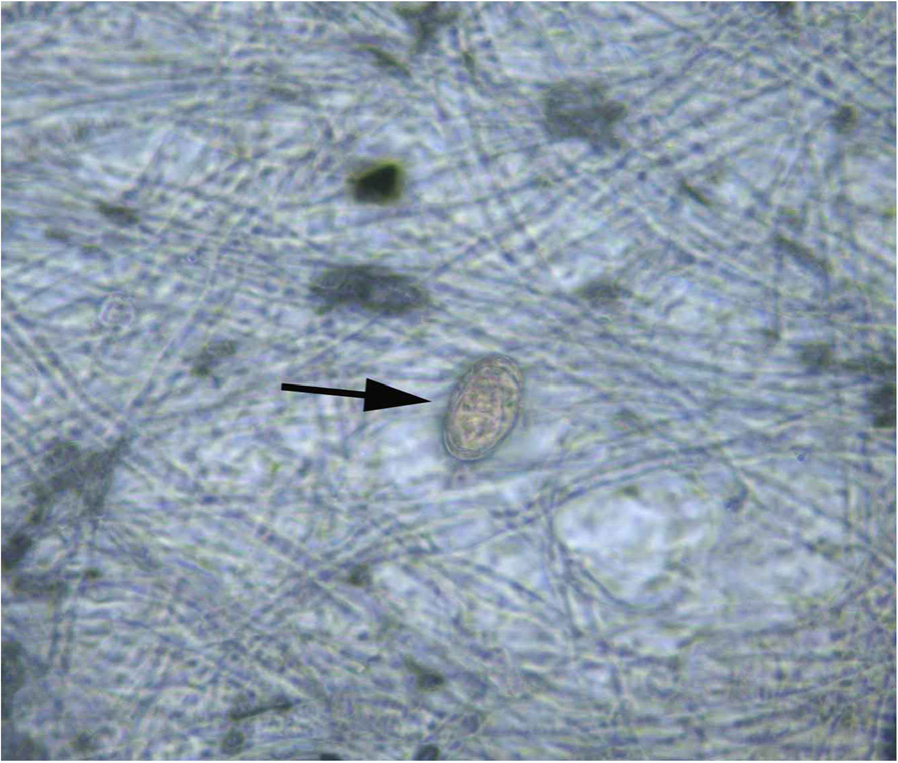
**Microscopic examination of the urine sediment detects *S. haematobium *egg in the urine specimen.**

On March 16, 2012, the patient reported significantly reduced white membrane-like substances in the urine and disappearance of gross hematuria; however, microscopic examination of the urine sediment still detected schistosome eggs (Figure 2). A routine blood test on April 25, 2012 in Xijing Hospital showed a WBC count of 10.85 × 10^9^/L, 1.1% eosinophil granulocyte, and 76.6% neutrophilic granulocyte. On May 4, 2012, the patient underwent re-examinations in Jiangsu Institute of Parasitic Diseases with disappearance of hematuria and membrane-like substances in the urine, the microscopic examination of the urine sediment revealed no schistosome eggs, and DDIA, IHA (titer, 1:5) and COPT tests were all negative.

*S. haematobium*, the etiologic agent of urogenital schistosomiasis, is the most prevalent anthropophilic schistosome species globally, which accounts for nearly half of the number of cases with schistosomiasis primarily in sub-Saharan Africa and the Middle East [11]. Although the symptoms are varied, the bulk of the morbidity and mortality of urogenital schistosomiasis can be ultimately attributed to the host immune response against *Schistosoma* eggs deposited within the walls of the urinary tract. This inflammation leads to compromise of urothelial integrity promoting urinary tract infections, hematuria, and protein-wasting; urothelial changes leading to carcinogenesis; and urinary tract fibrosis causing bladder dysfunction, obstruction, infection, and renal failure [12]. In addition, female genital schistosomiasis has been identified as a co-factor in Africa's AIDS epidemic, especially in Zimbabwe and Tanzania, where it was linked to a 3–4 times increased risk in acquiring HIV infection [13].

Currently, the gold standard for diagnosis of schistosomiasis haematobia still relies on the microscopic detection of parasite eggs present in urine specimens [14]; however, parasitological diagnosis in adults is difficult, particularly among persons who have chronic infections and pass only small numbers of eggs. Many immunodiagnostics and DNA-based diagnostics have been developed [15-20], which facilitate the detection of this neglected tropical disease. In the current study, three immunodiagnostic assays for the detection of antibodies against *S. japonicum*, including DDIA, IHA and COPT, were employed to detect *S. haematobium* infection, and the detection results coincided with the microscopic examination. It has been shown that DDIA using *S. japonicum* soluble egg antigen (SEA) can be used for the diagnosis of schistosomiasis mansoni and schistosomiasis mekongi [21,22]. Taken together, it seems that the currently available immunodiagnostics directed against *S. japonicum* antibody seem effective for the detection of *S. haematobium* infection. However, further studies are required to validate the performance of these immunodiagnostic assays in detection of schistosomiasis haematobia. In addition, in the absence of diagnostics for imported African schistosomiasis in China, a search for novel immunodiagnostics or molecular assays is urgently needed and should be given a high priority [23,24].

*S. haematobium* infection has been linked to bladder tumors, and the infection is reported to be responsible for the development of carcinoma of the urinary bladder [25-28]. In the current study, the imported case infected with *S. haematobium* was misdiagnosed as invasive urothelial carcinoma. The patient has not developed bladder tumors to date. Given that *S. haematobium* infection is identified as a factor in the pathogenesis of carcinoma of the urinary bladder [29], periodic health examination is suggested to detect bladder tumors at an early stage.

**Figure 3:**
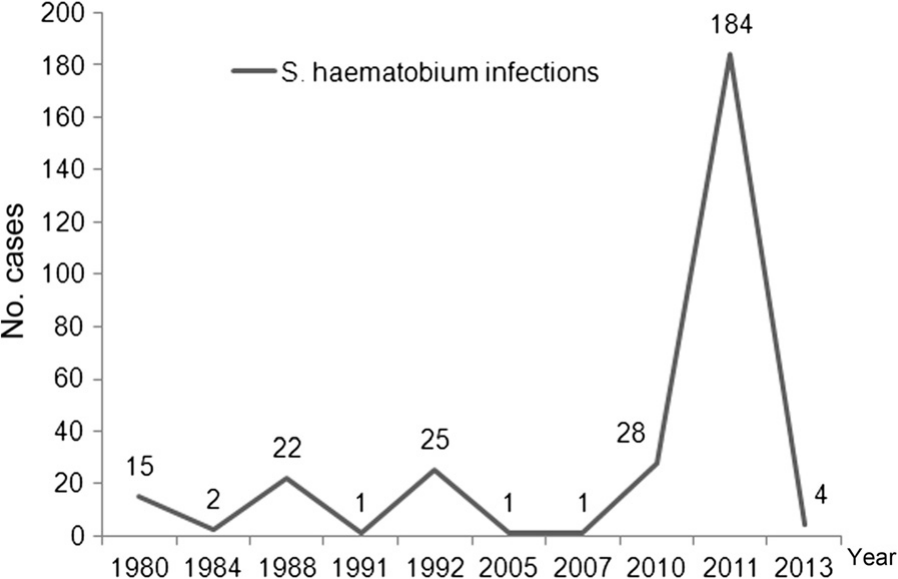
**Reported *S. haematobium *infections returning from Africa in China.**

Since the 1970s, when China initiated projects to aid African infrastructure development and sent engineering technicians and labor services to Africa, imported cases infected with *S. haematobium* have been continually reported in those returning from African countries. The quickening pace of integration of the global economy, the deepening collaboration between China and African countries and Chinese rapid economic development results in a recently gradual increase in the cases infected with *S. haematobium* returning from Africa to China (Figure 3). However, there is little knowledge on systematic epidemiological surveys of *S. haematobium* infections among laborers working in African countries till now. We summarized the features of imported cases with schistosomiasis haematobia according to the available data (Table 1). The low awareness and poor knowledge of prevention and control of *S. haematobium* infection results in the frequent misdiagnosis and makes imported schistosomiasis haematobia a truly “neglected tropical disease” in China. Unfortunately, the following problems increase the difficulty in the management of imported schistosomiasis haematobia in China (Table 2). It has been proved that *Biomphalaria straminea*, an intermediate host of *S. mansoni* introduced from Brazil, is able to survive, reproduce, and form new populations naturally in southern China [30]. Global warming may break the original landscape of schistosomiasis and create an appropriate condition for the survival and reproduction of the snail hosts *Bulinus* spp. in China [31]; therefore, it is considered that the risk of transmission of *S. haematobium* seems increasing [32], which must be paid much attention to avoid the occurrence of public health concern in China.

**Table 1:** Features of imported cases with schistosomiasis haematobia

1.	Most infections occur in young adults who are exported to African countries for labor services.
2.	Potential underestimation of actual number of cases. Most of the infections are detected at physical examinations, and few patients seek medical care; therefore, the actual number of patients with *S. haematobium* infections may be underestimated.
3.	High proportion of misdiagnosis. The major clinical manifestations of schistosomiasis haematobia involve hematuresis, bladder irritation, and urinary tract obstruction, which are often misdiagnosed as sexually transmitted diseases, cystitis, tuberculosis and tumors due to the lack of knowledge on diagnosis of the disease in Chinese clinicians.
4.	The cases are widely distributed in China, and have a high mobility [33].

**Table 2:** Problems currently present in the control of imported schistosomiasis haematobia

1.	Lack of sound multi-sector collaborations.
2.	No national criteria for the diagnosis and treatment of imported schistosomiasis haematobia.
3.	Lack of diagnostics for the detection of imported cases with *S. haematobium* infection.
4.	The exporting laborers have little knowledge on prevention and control of *S. haematobium* infections, and they lack active pursuit of medical care even if infected.
5.	Medical professionals lack awareness, diagnosis and treatment experiences as well as techniques regarding *S. haematobium* infection.

**Figure 4:**
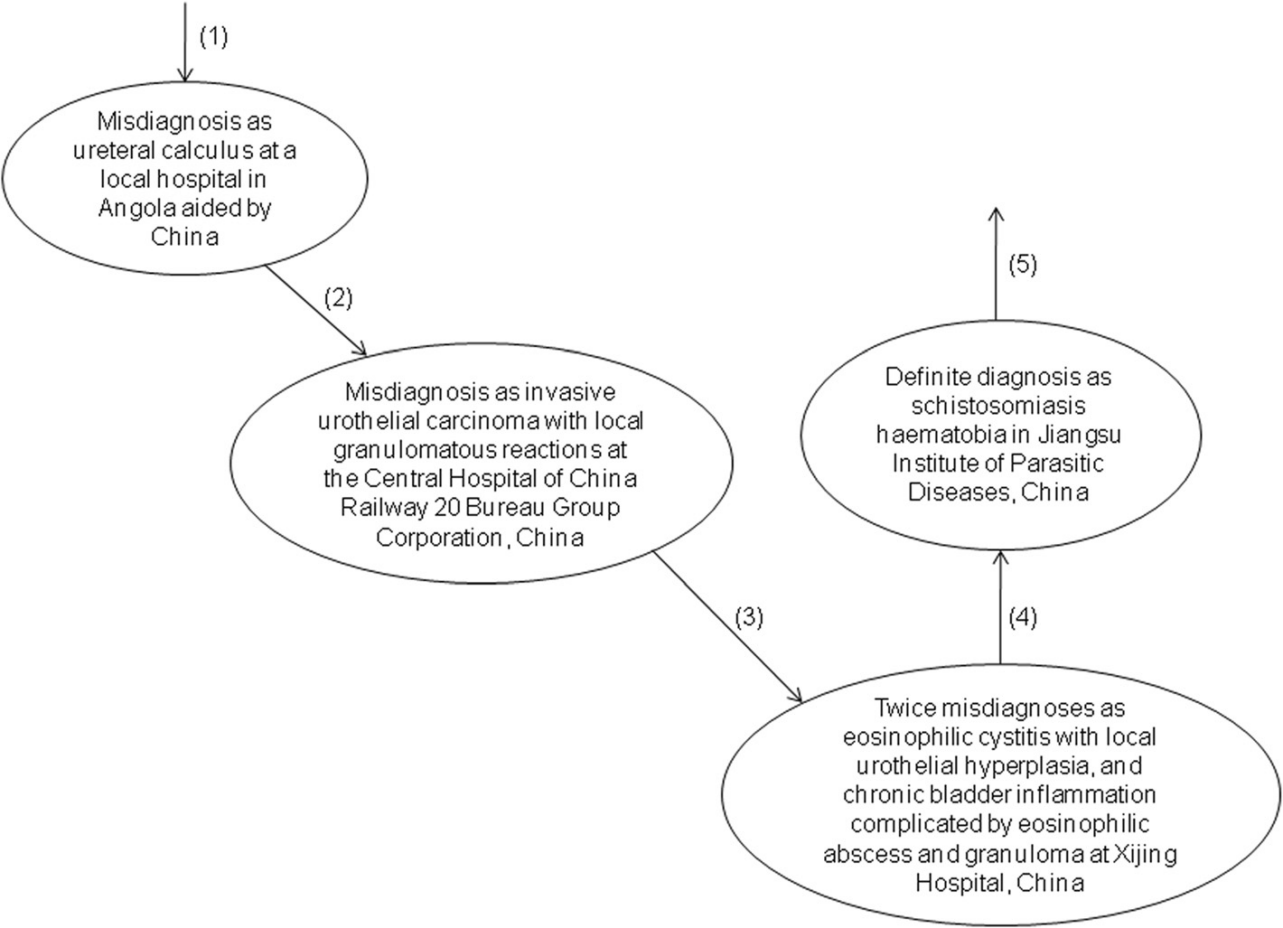
The misdiagnosis experience for the case.

In this study, the patient underwent multiple misdiagnoses before the definite diagnosis (Figure 4) and received several treatments including surgery in several hospitals. It is considered that the low awareness of this neglected tropical disease and the lack of knowledge and experiences on diagnosis and treatment causes the misdiagnosis, and the duration from admission to definite diagnosis is over one year. The multiple invasive examinations and treatments cause huge physical injuries and high economic burden. The following countermeasures are therefore proposed to improve the management of imported schistosomiasis haematobia (Table 3), and a surveillance-response system is required to monitor and respond the import of cases with schistosomiasis haematobia.

**Table 3:** Countermeasures to improve the management of imported schistosomiasis haematobia in China

1.	Strengthening multi-sector collaborations and the detection at the entry-exit inspection and quarantine sections, and involvement of schistosomiasis diagnosis in the routine physical examinations for worker returning from Africa.
2.	Development of national criteria for the diagnosis and treatment of imported schistosomiasis haematobia.
3.	Assessing the performance of immunodiagnostics directed against *S. japonicum* antibodies in detection of *S. haematobium* infection, and development of novel immunodiagnostics or molecular assays for the detection of *S. haematobium* infection.
4.	A systematic survey of the labor export to the schistosomiasis haematobia-endemic areas, and an evaluation of the true infections.
5.	Strengthening the training on knowledge about schistosomiasis haematobia among medical professionals, including diagnosis and treatment.
6.	Improving the access to health education pertaining to schistosomiasis status, prevention and control as well as international travel healthcare among those moving to Africa due to work, business or travel, so as to enhance their self-protection awareness and active pursuit of medical care if infected.

## Conclusions

As *S. haematobium* is not indigenous to China, most Chinese doctors and medical technicians are unfamiliar with this introduced parasitic disease, therefore, they need to increase the awareness of its existence when they encounter persons who have visited or resided in endemic areas, and the techniques for detection of the parasite, so as to reduce missing diagnosis and misdiagnosis. In addition, health education should be performed among those who will go to the endemic areas to improve their knowledge and awareness on prevention and control of schistosomiasis haematobia, thereby reducing the risk of exposure to the infested freshwater. It is considered that the management of imported schistosomiasis haematobia should be improved in China based on the lessons from this case undergoing multiple misdiagnoses.

## Competing interests

The authors have declared no competing interests exist.

## Authors’ contributions

HYH, WW and YSL conceived and designed the study; HYH, GQC and FT performed the detection and the follow up, and collected the clinical data. HYH and WW prepared the first draft of the manuscript; YSL provided strategic advice and assisted with editing of the manuscript. All authors read and approved the final version of the manuscript.
